# Epilepsy-induced heart damage and sudep risk: ion channelopathies, calcium homeostasis, and the therapeutic potential of resveratrol

**DOI:** 10.1007/s00210-026-05557-6

**Published:** 2026-06-10

**Authors:** Nilufer Akgun-Unal, Omer Unal, Emre Soner Tiryaki, Ahmet Akkoca, Seda Simsek, Elif Gulbahce-Mutlu, Mustafa Ayyildiz

**Affiliations:** 1 Department of Biophysics, Faculty of Medicine, Ondokuz Mayis University, Samsun, Türkiye; 2 Department of Physiology, Faculty of Medicine, Samsun University, Samsun, Türkiye; 3 Department of Physiology, Faculty of Medicine, Ondokuz Mayis University, Samsun, Türkiye; 4 Department of Biophysics, Faculty of Medicine, Necmettin Erbakan University, Konya, Türkiye; 5 Department of Histology and Embryology, Faculty of Medicine, Selcuk University, Konya, Türkiye; 6 Department of Medical Biology, Faculty of Medicine, KTO Karatay University, Konya, Türkiye

**Keywords:** Epilepsy, SUDEP, Heart dysfunction, Resveratrol, SIRT1, Ca^2+^channels

## Abstract

Epilepsy is a worldwide health issue associated with cardiac-related conditions and Sudden Unexpected Death in Epilepsy (SUDEP). The pathophysiology of SUDEP involves structural fibrosis of the myocardium, a calcium imbalance, and dysfunction of the autonomic nervous system. The objective of this investigation was to examine cardiac damage resulting from epilepsy triggered by the Pentylenetetrazol (PTZ) kindling rat model at a dosage of 35 mg/kg administered intraperitoneally, and to assess the therapeutic benefits of Resveratrol (RSV) at a dosage of 5 mg/kg. Cardiac function was evaluated using isolated papillary muscle recordings. Damage to the structure was assessed by Sirius Red staining and general tissue morphology. Molecular analyses encompassed qPCR (SERCA2a, CACNA1G, HCN2, CAS3/9) and immunofluorescence (SIRT1, Caspase-3, S100α) in conjunction with the total Ca^2^⁺ content. PTZ kindling enhanced seizure severity and overall spike number on ECoG. Functionally, contractile force (CF), contractile power (AUC), and contraction/relaxation velocities (± dF/dt_max_) were decreased in the PTZ group compared to the Sham group at 3, 4, and 5 Hz frequencies. Contraction duration (CT) was significantly prolonged at 5 Hz, whereas no significant change was observed in relaxation time (RT). Histopathology revealed degeneration and substantial myocardial scarring. At a molecular level, PTZ decreased SERCA 2a mRNA expression (*p* < 0.0001) while increasing total calcium content (*p* < 0.0001) and pro-apoptotic markers (Caspase-3/9). Furthermore, the protective SIRT1 protein signal intensity was substantially reduced. RSV therapy resulted in a reduction of seizure severity by 2.54 ± 0.13 and also ameliorated cardiac function. Mechanistically, RSV recovered expression of SERCA2a (*p* < 0.0001), reduced fibrosis/apoptosis, and reactivated the anti-apoptotic SIRT1 pathway. These findings show that RSV ameliorates epilepsy-induced cardiac injury and has the potential to serve as an effective supportive treatment against SUDEP-related heart dysfunction by regulating calcium levels and activating anti-apoptotic pathways.

**Supplementary Information:**

The online version contains supplementary material available at 10.1007/s00210-026-05557-6.

## Introduction

A group of chronic neurological disorders known as epilepsy has a complex cause and is one of the most prevalent serious brain disorders, affecting more than 70 million people globally (Thijs et al. 2019). This condition is typically marked by repeated seizures caused by a disparity between the brain's excitability and inhibitory mechanisms within the central nervous system (CNS). Seizures can impact specific brain systems or begin in a particular area and then spread to include numerous cortical and subcortical circuits (Steinlein 2004). Sudden unexpected death in epilepsy (SUDEP) is a major cause of mortality among patients with epilepsy. Although its incidence is relatively low in patients with well-controlled seizures, the risk increases dramatically in young adults with drug-resistant (refractory) epilepsy. In this specific high-risk population, SUDEP accounts for up to 18% of all deaths (Alzahrani et al. 2023). Furthermore, the underlying cardiac mechanisms of SUDEP have been shown to include autonomic nervous system dysfunction, lethal arrhythmias secondary to QT interval abnormalities, genetic overlap with cardiac channelopathies, and structural myocardial fibrosis (Alzahrani et al. 2023; Chahal et al. 2020; Poddi et al. 2025). In the vast majority of reported cases, generalized tonic–clonic seizures are the leading risk factor for SUDEP. There is substantial evidence documenting that effective treatment with the right antiepileptic drugs (AEDs) can prevent SUDEP (Aurlien et al. 2016). Altered seizures can disrupt the autonomic nervous system's command over cardiorespiratory function, resulting in hypoventilation, apnea, and cardiovascular collapse (Devinsky et al. 2016; Manolis et al. 2019). The exact cause of SUDEP remains unclear, but the majority of sudden cardiac deaths are attributed to underlying cardiac issues (Priori et al. 2015). Seizures have been associated with various arrhythmias that can occur during the seizure or afterwards; the most frequently reported are ictal asystole, bradycardia, and AV block. The presence of cardiovascular disease seems to contribute substantially to higher mortality rates in individuals with epilepsy than in the general population. Research has shown that individuals with epilepsy are more likely to have structural heart problems than people who do not have epilepsy (Shmuely et al. 2017). Evidence of heightened cardiac autonomic stimulation has been observed in SUDEP patients, particularly during epileptic seizures that occur during sleep. Repeated stimulation can lead to structural heart damage via myocardial fibrosis, as seen in SUDEP patients (Jansen and Lagae 2010). Studies using animal models of epilepsy have discovered that cardiac hypertrophy is linked to changes in the autonomic nervous system resulting from seizures, indicating that seizures could structurally impact the heart (Naggar et al. 2014).

Research in animal models has demonstrated that resveratrol (RSV) possesses a variety of additional health benefits, such as anti-ischemic, antiviral, antioxidant, and anti-inflammatory effects (Akgun-Unal et al. 2023a, b; Belguendouz et al. 1997; Campagna and Rivas 2010; Jang et al. 1999; Kraft et al. 2009; Manna et al. 2000; Robich et al. 2010; Sato et al. 2000; Sun et al. 2010). Multiple studies of cell cultures and animal models with neurodegenerative diseases/brain injury have shown that RSV is a powerful neuroprotective compound (Bastianetto et al. 2000; Baur and Sinclair 2006; Han et al. 2004; Jang and Surh 2003; Liu et al. 2011). Our intention here was to examine the therapeutic impact of RSV on any alterations in cardiac contractility related to SUDEP in a rat model of epilepsy that mimics seizure activity. In this established tool, seizure susceptibility is chemically induced by intraperitoneal injection of pentylenetetrazole (PTZ), a GABAA receptor antagonist known to cause a persistent increase in seizure susceptibility with minimal neuronal damage (Huang et al. 2001). In our study, we specifically investigated SERCA 2a, CACN A1G and HCN2 channels because they play critical roles in cardiac excitation–contraction coupling and pacemaker activity. Alterations in the expression of these specific targets may constitute the cellular basis of cardiac channelopathies developing secondary to epilepsy.

## Material and methods

### Animals

A total of 32 adult male Wistar albino rats weighing 250–300 g were obtained from the Ondokuz Mayıs University Laboratory Animal Application and Research Center. Only male rats were used in this study to eliminate the confounding effects of female hormonal fluctuations (i.e., the estrous cycle) on seizure susceptibility and baseline cardiovascular parameters. The rats were housed under standard laboratory conditions (22 ± 2 °C, 50–60% humidity, and a 12-h light/dark cycle) and provided with ad libitum access to standard pelleted rodent diet and tap water. All experimental procedures were approved by the Local Ethics Committee on Animal Research of Ondokuz Mayıs University (Approval No: 2024/45) and were carried out strictly in accordance with the National Institutes of Health (NIH) Guide for the Care and Use of Laboratory Animals. Furthermore, this study was designed and reported in accordance with the ARRIVE guidelines.

### Chemicals and experimental design

PTZ (P6500, Sigma-Aldrich, St. Louis, MO, USA) and RSV (R0071, TCI, Tokyo, Japan) were dissolved in isotonic saline (0.9% NaCl) and prepared fresh on the day of administration.

**Fig. 1:**
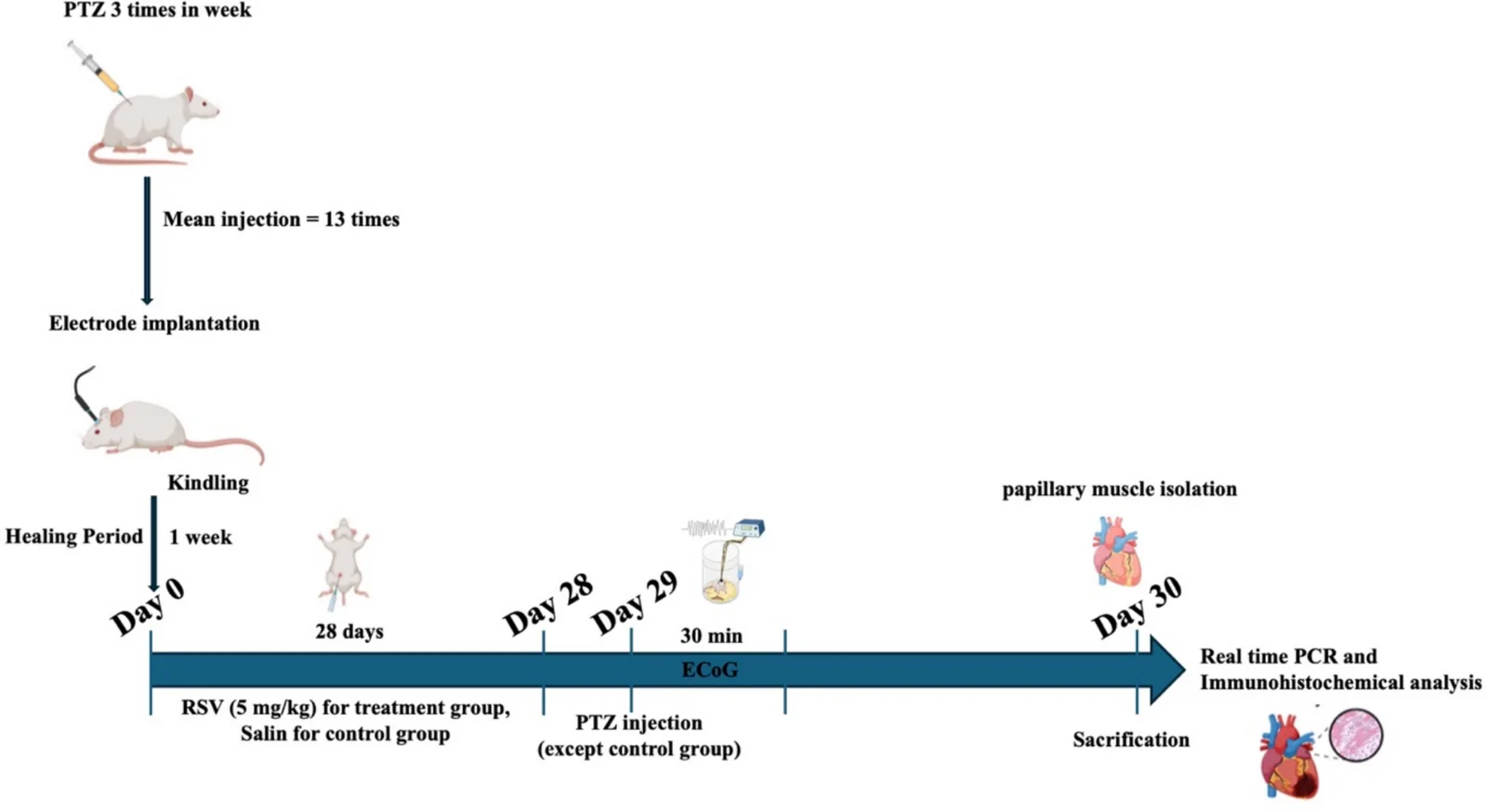
Time scale of the experiments

Rats were randomly assigned as follows:

- Sham (*n* = 8): During the PTZ injection process for the PTZ and PTZ + RSV groups, physiological saline was injected intraperitoneally (i.p.) in the same volume as PTZ, followed by electrocorticography (ECoG) electrode implantation. After a one-week healing period (7 days), the RSV group received intraperitoneal injections of physiological saline containing RSV as the solvent at the same volume and for the same duration as the RSV group for 4 weeks.
- RSV (*n* = 8): The RSV group underwent the same procedures as the sham group until the end of the healing period, followed by i.p. RSV injection at 5 mg/kg × 28 days (Akgun-Unal et al. 2023a, b).
- PTZ (*n* = 8): In this group, rats received i.p. injections of 35 mg/kg PTZ three times a week (Monday, Wednesday, and Friday) until kindling occurred (average 14 injections) (Günaydın et al. 2020). After the healing period, RSV solvent physiological saline was administered intraperitoneally for the same volume and duration as RSV.
- PTZ + RSV (*n* = 8): After the same procedures as the PTZ group were performed and the healing period was completed, RSV was administered intraperitoneally at the same dose and duration as the RSV group. An overview of the entire experimental design is presented in Fig. 1.

### PTZ-induced kindling model

The PTZ-induced kindling model was established as previously described (Zhu et al. 2016). Rats were administered a subthreshold dose of PTZ (35 mg/kg, i.p.) three times a week (Monday, Wednesday, and Friday) for 1 month until seizures began to develop. Following each injection, the rats were individually confined to a transparent plexiglass enclosure, and their convulsive behaviour was recorded on video for 30 min. The recordings were examined without bias for seizure stage, seizure onset latency, and seizure duration. The animals' seizure behaviors will be scored according to the study by Fisher and Kittner (Fischer and Kittner 1998). The stages of seizure activity are defined by the following scoring: Stage 0, no seizure activity; Stage 0.5, weak head shaking; Stage 1, twitching in the ears, face, and eyelids; Stage 1.5, mild clonic activity in the forelimbs; Stage 2, myoclonic body jerks and clonic convulsions in the forelimbs without elevation; Stage 2.5, frequent clonic convulsions in the forelimbs with brief elevation; Stage 3, severe clonus (lasting ≥ 10 s) in both forelimbs with full elevation; Stage 3.5, severe bilateral forelimb clonus with elevation and falling; Stage 4, episodes of elevation and falling, or jumps with generalized clonic convulsions; Stage 4.5, generalized clonic-tonic seizures with loss of righting reflex; Stage 5, generalized clonic-tonic seizures and status epilepticus (lasting ≥ 2 min). Rats were considered to have seized when they exhibited stage 4 or 5 after three consecutive PTZ injections (Chen et al. 2017).

### Electrocorticography (ECOG) recordings

Rats that had been made reactive by being kindled were anesthetized using a combination of 90 mg/kg ketamine and 10 mg/kg xylazine before being placed in a stereotaxic apparatus supplied by Stoelting Company, USA. A skin incision was made to locate the Bregma following shaving of the relevant area. Three 1-mm wide holes were drilled into the skull using an OmniDrill35 dental motor from WPI in Korea, at the specified coordinates, without compromising the meninges. Three screws were positioned at specified points: one in the frontal cortex (4 mm in front of and 3 mm to the right of Bregma), another in the occipital cortex (4 mm behind and 3 mm to the right of Bregma), and a reference electrode at 4 mm behind and 3 mm to the left of Bregma. The bipolar electrode was then secured around the screws with dental acrylic (Günaydın et al. 2020). Following surgery, all experimental groups including the Sham controls received buprenorphine hydrochloride (0.1 mg/kg, intramuscularly) for analgesia and ampicillin (50 mg/kg, intraperitoneally, twice daily for three consecutive days) for infection prophylaxis, ensuring that surgical stress and medication effects were standardized across all animals. Twenty-four hours after the last injection in each experimental group, the rats were housed individually in separate enclosures and linked to a data acquisition system comprising the ML870/P, PowerLab 8/30 from ADInstruments, Australia, and the ECoG recordings were transmitted to a digital storage device for further analysis using Labchart7 software over a 30-min period. Spike quantification was performed offline. Sharp waves that significantly exceeded the baseline ECoG activity’s amplitude and frequency thresholds were defined and counted as spikes, and the total number of spikes was determined.

### Isolation of the myocardial papillary muscle

While the rats were under anesthesia, their hearts were quickly removed and placed in Krebs solution (135 mM NaCl, 5 mM KCl, 2.5 mM CaCl₂, 1 mM MgSO₄0.7H₂O, 1 mM NaH₂PO₄0.2H₂O), which had been pre-gassed with a mixture of 95% O₂ + 5% CO₂ and adjusted to pH 7.40. It was then placed in a Petri dish with a Slygard gel-coated bottom containing the same solution and fixed in this solution with a small needle so that the hearts were visible from the dorsal region. An incision was made in the ventricular wall from the atrioventricular valve level toward the apex, and the ventricle was opened at this site. The papillary muscles were isolated using microsurgical scissors to avoid any pressure on the hearts (Akgun-Unal et al. 2023a, b, 2025).

In this study, contraction recordings from the myocardial papillary muscle were made as isometric contractions. Isolated papillary muscles were secured at both ends with 6/0 silk thread. Tissues were placed in an isolated organ bath (MAY IOBS99 Isolated Tissue Bath and Circulator, Commat Ltd.) containing 30 ml of fresh Krebs solution. The temperature of the Krebs solution was maintained at 33 °C using a heating jacket in the organ bath, a MAY WBC 3044 Water Bath and Circulator from Commat Ltd. A metal hook on one of the threads attached to the papillary muscles was linked to a manually controlled micromanipulator, while the other hook was attached to a force transducer (FDT05 Force Displacement Transducer, Grass Co.), with muscle tension being adjusted to achieve the maximum contraction. Square-wave stimuli exceeding the maximum threshold were administered through a field stimulator, specifically a MAY ISO 150-C Stimulus Isolation Power Supply, and directed at the papillary muscle via an electrode positioned between the anode and cathode terminals. Data for all contractions were collected at a sampling rate of 1 kHz on a hard disk via an analog-to-digital converter (MP36 Four channel data acquisition unit, Biopac System Inc.) and its associated software (BSL PRO, version 4.1.1, developed by Biopac System Inc.). After the completion of the contraction recordings, the papillary muscles were removed from the organ-bath, their attached strings were untied, and their weights were measured dry using an Ohaus PA2224C balance with a precision of 0.1 mg.

Following the placement of the papillary muscle in the organ bath, square-wave stimuli with a fundamental frequency of 0.2 Hz, 10–15 V (supramaximal), and a duration of 2 ms. To ensure optimal physiological conditions, the Krebs solution was replaced every 15 min during the 1-h stabilization period. Contractions were monitored simultaneously. Frequency-dependent contraction responses were evaluated once the contraction amplitudes had stabilized at the end of the 1-h period. Supramaximal square-wave stimuli (10–15 V, 2 ms duration) were applied at 1, 2, 3, 4, and 5 Hz. For each frequency, 20 peak values were recorded before proceeding to the next level, and the contraction curves were tracked accordingly (Ahmet et al. 2023; Pieske et al. 1999).

### Total RNA isolation and qPCR analysis

Total RNA was extracted from cardiac tissue using a commercial kit (Bio Basic; Canada) according to the manufacturer's instructions. RNA purity and concentration were measured spectrophotometrically using a microplate reader (Multiskan SkyHigh Microplate, Thermo Fisher, USA), and electrophoretic analysis was used to confirm RNA integrity.

cDNA was synthesized using a commercially available reverse transcription kit (High Capacity cDNA Reverse Transcription Kit, Catalog No: 4368813, Thermo Fisher, Lithuania) by the ProFlex PCR System (Thermo Fisher, USA) according to the manufacturer's protocol.

The resulting cDNA was amplified using primers designed to target the following genes: calcium voltage-gated channel subunit alpha1 G (CACN A1G), hyperpolarization-activated cyclic nucleotide-gated potassium and sodium channel 2 (HCN2), ATPase sarcoplasmic/endoplasmic reticulum Ca^2+^ transporter 2 (SERCA 2a), Caspase 3 (CAS3), Caspase 9 (CAS9), and SIRT1. All reactions were performed in triplicate in 20 μl volumes using FastStart Essential DNA Green Master mix (Cat. No: 06 402 712 001, Roche, Switzerland).

The primers used for mRNA expression analysis of the target genes were synthesized by the manufacturer (Oligomer, Turkey). GAPDH was used as an endogenous control (Table 1).

**Table 1:** Primers used for qPCR

Gene	Primer Sequence	
CACN A1G	Forward	5’-CATGCCACCTTTAGGAACTTTG-3’
	Reverse	5’-CGGAGGGTGTCCTTCATAATAC-3’
HCN2	Forward	5’-GGTGGACTACATCTTCCTCATC-3’
	Reverse	5’-CAGACTGAGGATCTTGGTGAAA-3’
SERCA2a	Forward	5’-CAGAGAGACGCCTGCTTAAAT-3’
	Reverse	5’-GTTCACACCATCACCAGTCATA-3’
CAS3	Forward	5’-CCACGGAATTTGAGTCCTTCT-3’
	Reverse	5’-CCACTCCCAGTCATTCCTTTAG-3’
CAS9	Forward	5’-AGCCAGATGCTGTCCCATAC-3’
	Reverse	5’-CAGGAGACAAAACCTGGGAA-3’
SIRT1	Forward	5’-CATAGGTTAGGTGGCGAGTATG-3’
	Reverse	5’- GTTGGTGGCAACTCTGATAAATG-3’
GAPDH	Forward	5’-AACTCCCTCAAGATTGTCAGCAA-3’
	Reverse	5’-GGCATGGACTGTGGTCATGA-3’

The reaction mixture was formulated by combining 10 μl of Master Mix containing enzyme, dNTP, Mg, buffer, and water, 5 μl of cDNA, 1 μl of forward primer, 1 μl of reverse primer at a concentration of 10 pmol/μl, and 3 μl of RNase-free water. Following primer optimization, the PCR conditions were set at: an initial denaturation step at 95 °C for 10 min, denaturation at 95 °C for 10 s, annealing at 60 °C for 10 s, and polymerization at 72 °C for 10 s, for a total of 45 cycles. The melting curve was analyzed in a single cycle, comprising: 15 s at 95 °C, 1 min at 65 °C, and 15 s at 97 °C. All reactions were conducted using a QuantStudio 3 PCR machine, which is manufactured by Thermo Fisher and based in the USA. The obtained data were analyzed using the comparative Ct (ΔΔCt) method of relative quantification and prepared for comparison between groups (Livak and Schmittgen 2001).

### Calcium assay

The calcium content in heart tissue was measured colorimetrically using a commercial kit (Elabscience, E-BC-K103-M). This measurement was performed using 20 mg of tissue according to the manufacturer’s instructions. Measurements were taken at 610 nm using a microplate reader (Multiskan SkyHigh Microplate, Thermo Fisher, USA) at the end of the reaction. The results are expressed in mmol/gprot.

### Histopathological studies

Tissue from the hearts of animals that were sacrificed was removed, then washed with PBS and fixed in 4% paraformaldehyde for 24 h. After fixation, the tissues were preserved in a 30% sucrose solution and then embedded in a cryomatrix before being sectioned at a thickness of 5 µm using a cryostat.

For overall histological assessment, sections were stained with Hematoxylin & Eosin (H&E). Myocardial cells integrity, myocardial degeneration, interstitial inflammation, and vascular congestion were assessed using H&E staining. Additionally, Hematoxylin & Eosin (H&E) stained sections of cardiac tissue were semi-quantitatively evaluated to determine the degree of myocardial damage. Histopathological examination was performed based on parameters such as myocardial degeneration, inflammatory cell infiltration, and vascular congestion. Each parameter was scored from 0 to 3 according to the precise extent and severity of the tissue damage: 0: none (normal morphology); 1: mild (focal damage affecting < 25% of the examined tissue area); 2: moderate (multifocal damage affecting 25–50% of the tissue area); and 3: severe (diffuse damage affecting > 50% of the tissue area) (Gibson-Corley et al. 2013). All sections were examined by two independent observers blinded to group information (Supplementary Table 1).

Fibrosis evaluation was also performed using Sirius Red staining (Poddi et al. 2025). Collagen accumulation was evaluated using a semi-quantitative scoring technique. Tissues examined under a light microscope were semi-quantitatively graded using a scale of 0–4 for interstitial fibrosis and 0–3 for perivascular fibrosis (Biernacka and Frangogiannis 2011; Weber and Brilla 1991).

### Immunofluorescent labeling

Immunofluorescence labeling was performed to evaluate specific cellular responses in the heart tissue. Tissues were incubated in PBS containing 5% BSA and 0.2% Triton X-100 at room temperature for 20 min. After washing with PBS, the tissue was incubated with a protein-blocking solution at 37 °C for 30 min. The blocking solution was removed using a pipette without washing. In this study, cell death was investigated by evaluating SIRT1 protein expression**,** which is a molecular indicator of the cellular stress response and protective mechanisms associated with resveratrol, using anti-SIRT1, and caspase-3 activity, which is involved in the final stage of apoptosis, using anti-Caspase-3. Cardiac cell damage and its relationship with calcium-binding protein were evaluated by S100α antibody labeling. The sections were then treated with goat anti-rabbit IgG (H + L) (656,111) and FITC (656,111) secondary antibodies. The sections were covered with DAPI-containing mounting medium. Procedures were performed in a dark environment by creating a humidity chamber. Sections were examined under an Olympus BX51 trinocular fluorescence microscope, and the areas were examined at 4x, 10x, 20x, and 40 × magnifications. Four random areas were selected from each at 40 × magnification, and images were recorded with a DP72 camera. The ratio of anti-SIRT1, anti-S100α, and anti-caspase 3 labeled areas to the total area in the recorded images was calculated using the Image J program (National Institutes of Health, Bethesda, MD, USA).

### Statistical analysis

All data obtained from the study are presented as mean ± standard error (SEM). The normality of the data distribution was evaluated using the Shapiro–Wilk normality test. For statistical comparisons between groups, since seizure behavior scoring data (FMJ latency, seizure phase, and number of spikes) were compared only between two groups with observed seizures (PTZ and PTZ + RSV), an unpaired t-test was used. For more than two groups, data conforming to a normal distribution were analyzed using One-way ANOVA and Tukey post hoc test, while data not conforming to a normal distribution were analyzed using Kruskal–Wallis post hoc test Dunn's. A p-value < 0.05 was considered statistically significant. All statistical calculations and graph preparation were performed using GraphPad Prism (version 9.5.0, Boston, MA, USA) and Microsoft Office 365 (Microsoft Corp., Redmond, WA, USA) software.

## Results

### Effects of resveratrol on first myoclonic jerk latency, seizure, and total spike number

**Fig. 2:**
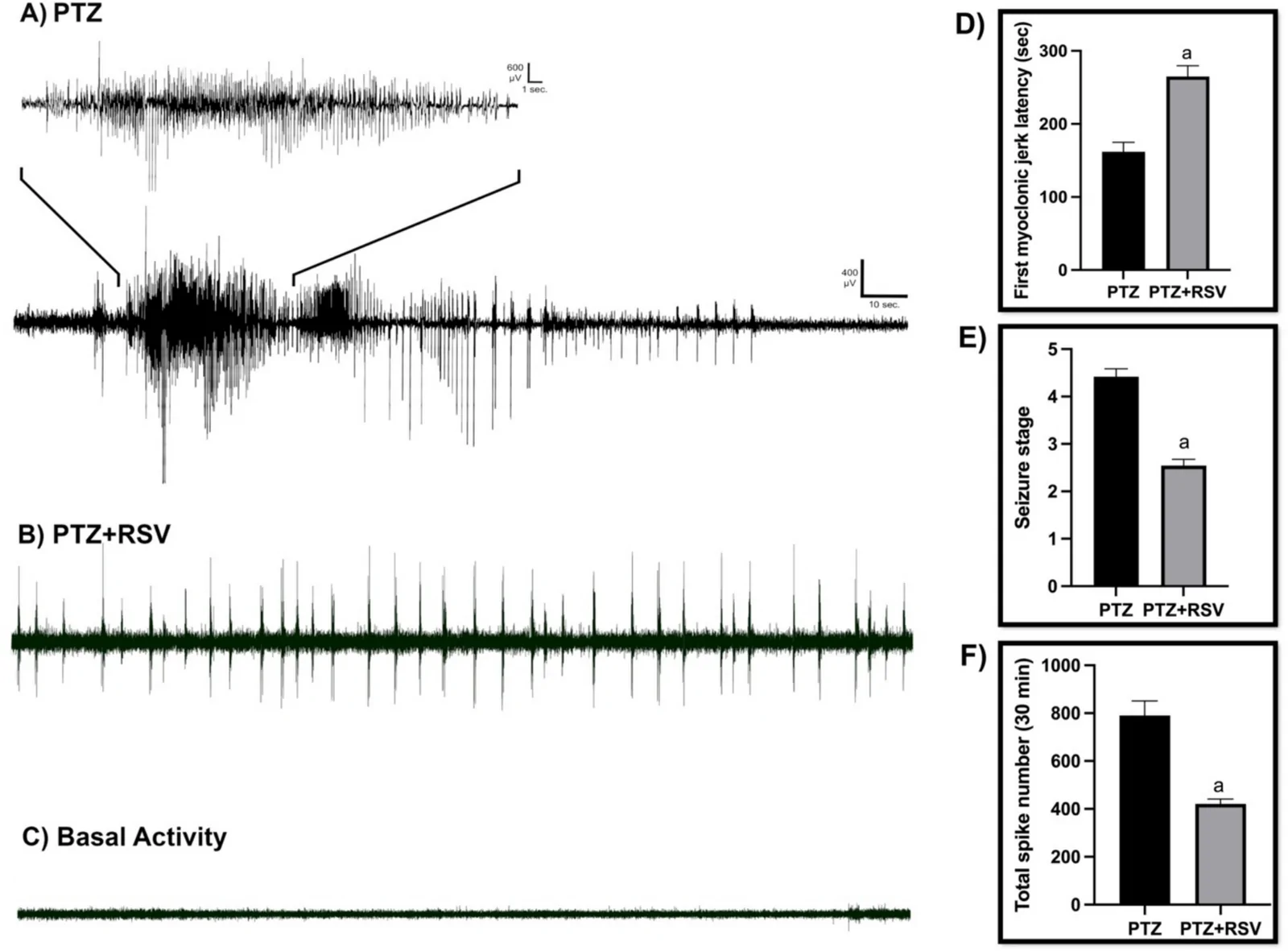
Representative ECoG recordings were taken after the last three PTZ administration. **A** Pentylenetetrazol (PTZ): Severe seizure activity after PTZ administration. **B** PTZ + RSV: A trace of improvement showing that resveratrol treatment significantly reduced the severity and duration of seizures. **C** Basal Activity (Sham/RSV): Since no difference in basal brain activity was observed between the Sham and RSV groups, a single recording representing both groups is presented. Effects of RSV (5 mg/kg) on seizure parameters, **D** First myoclonic jerk latency, **E** Seizure stage, **F** Total spike number. *p* values indicate unpaired *t*-test results. ^a^ vs. PTZ indicates statistical significance at the *p* < 0.05 level. Data are presented as mean ± SEM (*n* = 8)

ECoG recordings from all experimental groups showed no statistically significant difference between the Sham and RSV groups, exhibiting a normal brain activity pattern with low amplitude and high frequency. A single basal activity recording was used to represent both groups (Fig. 2C). However, the PTZ group exhibited high-amplitude and intense seizure activity (Fig. 2A). In contrast, the PTZ + RSV group exhibited a remarkable decrease in both the intensity and duration of seizures, and RSV exhibited an anticonvulsant effect against PTZ-induced neural overstimulation (Fig. 2B).

In the PTZ group, the first myoclonic jerk latency was 161.96 ± 12.79 s, the seizure stage was 4.41 ± 0.168, and the total spike number was 790.66 ± 60.34. Resveratrol administration increased the first myoclonic jerk latency (264.81 ± 14.90 s) (*p* = 0.0001). However, it decreased the seizure stage (2.54 ± 0.13) (*p* < 0.001) and total spike number (420.92 ± 20.59) (*p* < 0.0001) compared to the PTZ group (Fig. 2D, E, F).

### Effects of resveratrol on isometric contraction recordings from rat papillary muscles

**Fig. 3:**
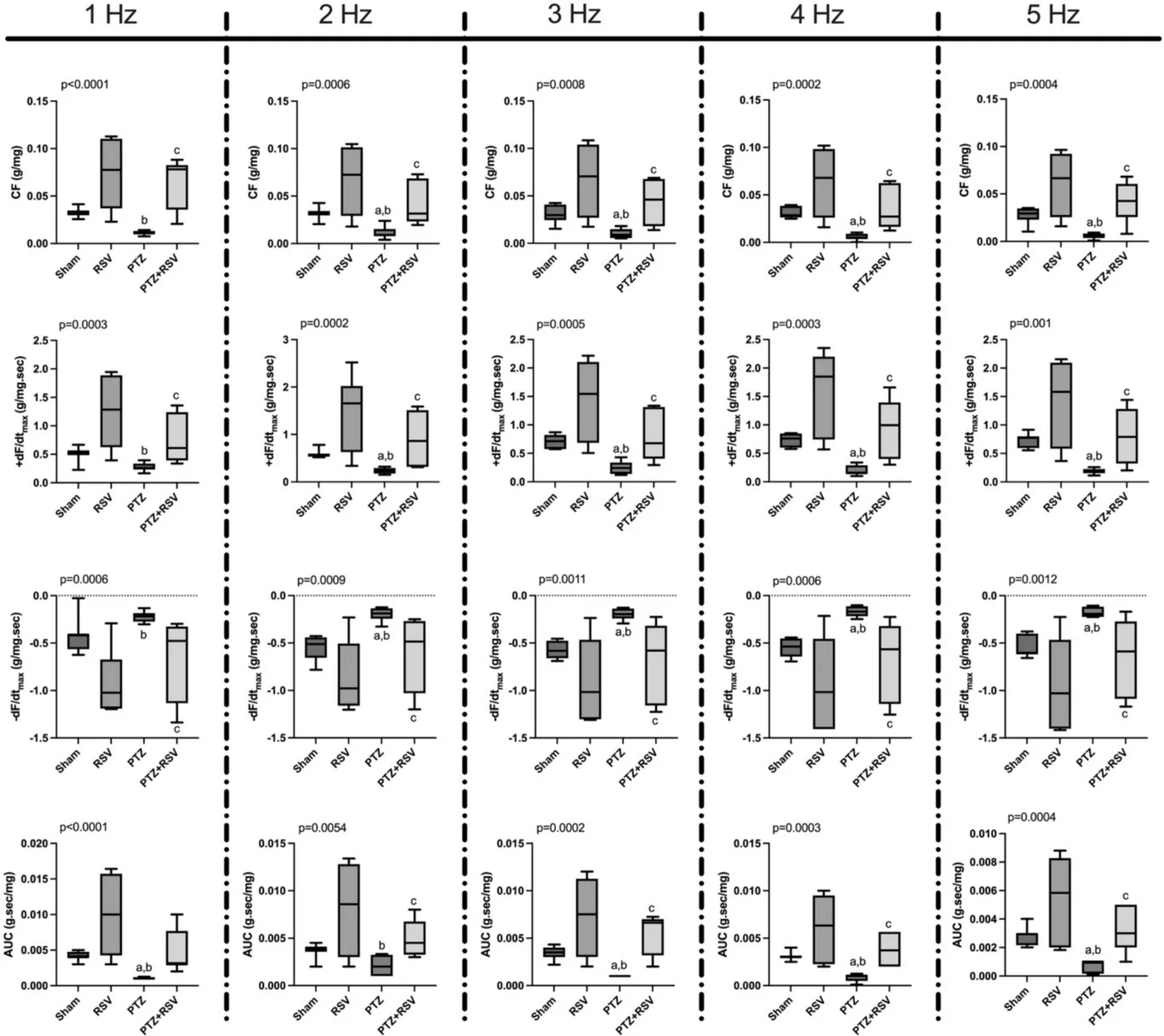
Comparison of parameters obtained from isometric contraction recordings of rat papillary muscles. Isometric contractility data were analyzed using the Kruskal–Wallis test followed by Dunn's multiple comparison test. ^a^ vs. Sham, ^b^ vs. RSV, ^c^ vs. PTZ indicate statistical significance at the *p* < 0.05 level

Isometric contraction recordings from isolated papillary muscles revealed the detrimental impact of PTZ-induced epileptogenesis on cardiac function and the restorative potential of RSV. Cardiac contractile performance was evaluated across a stimulation frequency range of 1 to 5 Hz (Fig. 3). Compared to the sham group, the PTZ-kindled rats exhibited a marked impairment in overall cardiac contractility, characterized by significant decreases in both Contraction Force (CF) and Contraction Power (AUC) across the majority of the tested frequencies. Treatment with RSV (5 mg/kg) effectively reversed these deficits; in the PTZ + RSV group, both CF and AUC values were significantly elevated compared to the PTZ group, successfully restoring contractile performance primarily at frequencies from 2 to 5 Hz. Similarly, the RSV-only group displayed significantly higher CF and AUC values than the PTZ group across all frequencies (Fig. 3).

Regarding contraction and relaxation kinetics, PTZ application significantly impaired both the maximum contraction rate (+ dF/dt_max_) and maximum relaxation rate (-dF/dt_max_) at frequencies between 2 and 5 Hz. Notably, RSV administration (PTZ + RSV group) effectively mitigated these kinetic deficits, yielding significant increases in both + dF/dt_max_ and -dF/dt_max_ across all frequencies when compared to the PTZ group (Fig. 3). Furthermore, the Contraction Time (CT) was significantly prolonged in the PTZ group compared to the sham group, particularly at the higher frequency of 5 Hz. This epilepsy-induced prolongation in CT was significantly attenuated following RSV treatment (PTZ + RSV group). In contrast, the Relaxation Time (RT) remained unaltered, with no statistically significant differences observed among any of the experimental groups (Supplementary Fig. 1).

### Effects of resveratrol on histopathological findings in rat heart tissue

**Fig. 4:**
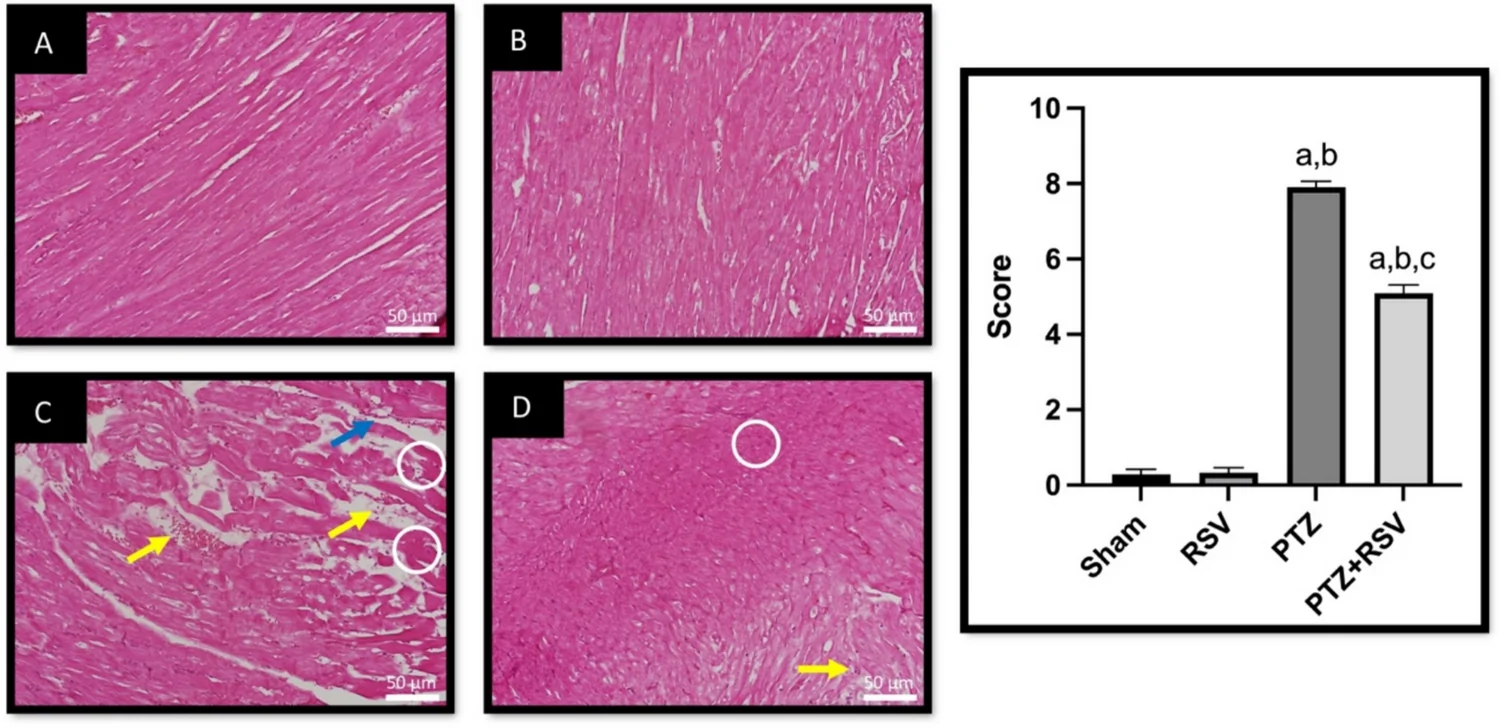
Histological examination of Hematoxylin & Eosin (H&E) stained heart tissue in PTZ-induced epilepsy rats. **A** Sham group: Myocardial fibers are neatly arranged, and cardiomyocytes have a normal structure. No inflammation or congestion in the interstitial tissue was observed. **B** RSV group: Tissue architecture appears similar to that of the Sham group, with cardiac myocyte nuclei being distinct and regular. Inflammatory cell infiltration was detected. **C** PTZ group: marked hyper-eosinophilia and myocyte degeneration are evident in the areas indicated by white circles. The yellow arrows indicate congestion at the capillary level, whereas the blue arrow indicates inflammatory cell infiltration between myocardial fibers. **D** PTZ + RSV group: Myocyte degeneration (white circle) appears to be reduced compared with the PTZ group. The vascular congestion indicated by the yellow arrow is mild. Interstitial inflammatory cell accumulation is markedly limited H&E, 20 × magnification. The graph shows the statistical results based on semi-quantitative scoring. p-values represent the One-way ANOVA result, and letters above the columns indicate statistical significance according to the Tukey post-hoc test results. ^a^ vs. Sham, ^b^ vs. RSV, ^c^ vs. PTZ indicate statistical significance at the *p* < 0.05 level. Data are presented as mean ± SEM

Histopathological examinations revealed that the myocardial fibers of the sham group heart tissue had normal morphology and parallel course, with distinct cardiomyocyte nuclei. No inflammation, edema, or vascular congestion was observed in the interstitial area. Cardiomyocyte placement and myocardial fiber integrity were preserved in the RSV group. There were no signs of inflammation or degeneration were observed in the interstitial area. Interstitial edema and inflammatory cell infiltration were observed in the myocardial tissue of patients with PTZ. In some areas, hyper-eosinophilic myocytes and fibers that had lost their nuclei, suggesting myocardial degeneration, were observed. Vascular congestion was observed at the capillary and small vessel levels. Muscle fiber integrity was preserved, myocardial degeneration and inflammatory infiltration were reduced, and vascular congestion was absent in the PTZ + RSV group (Fig. 4). These results suggest that resveratrol has a histological damage-reducing effect. According to H&E scoring results, a statistically significant increase (*p* < 0.05) was observed in the PTZ group compared with the sham group, whereas a statistically significant decrease was observed in the PTZ + RSV group compared with the PTZ group (Fig. 4).

**Fig. 5:**
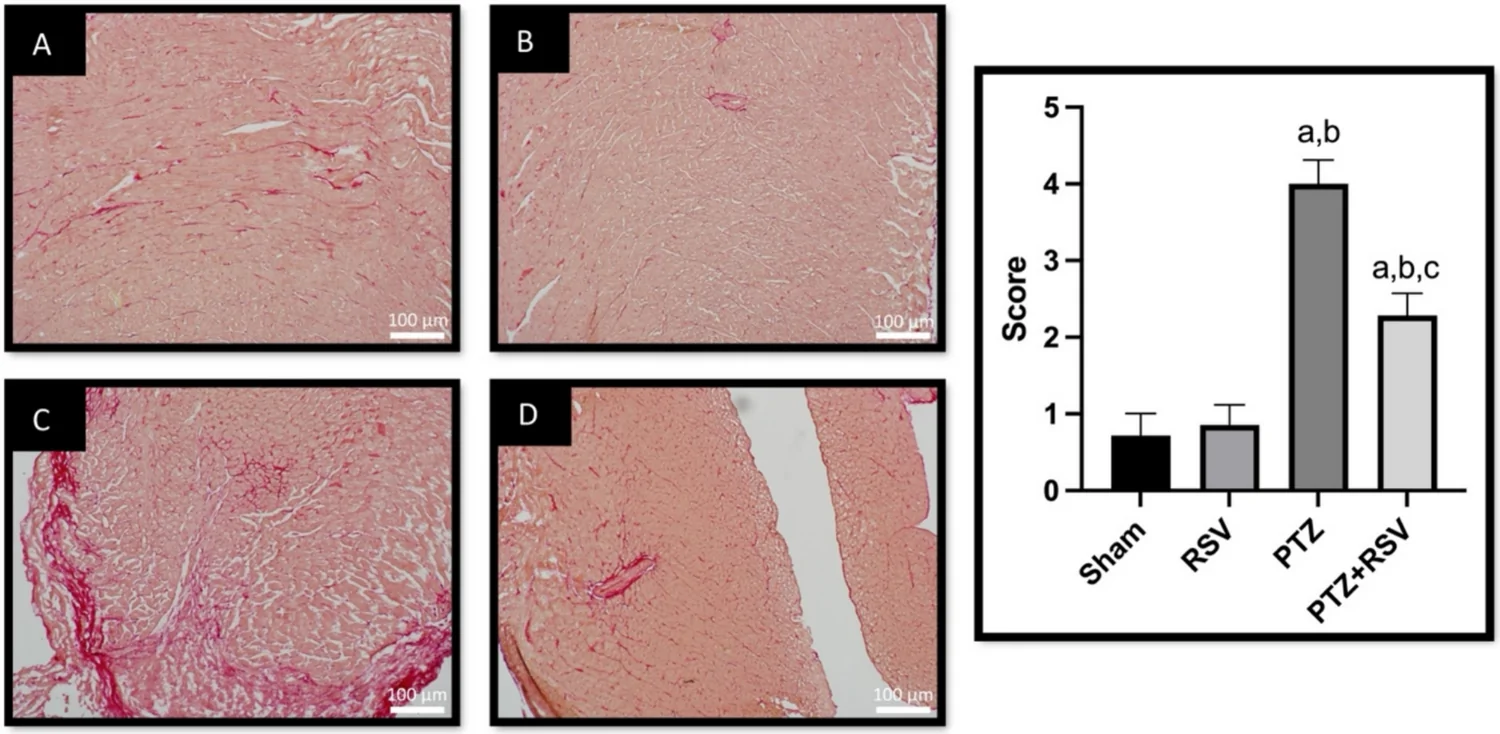
Evaluation of fibrosis in heart tissue using Sirius Red staining. **A** Sham group**:** Regular myocardial fibers and minimal collagen accumulation. **B** Resveratrol group: Low collagen levels and no fibrosis. **C** PTZ group: increased interstitial collagen accumulation and evident fibrosis. Wide gaps are observed between the myocardial fibers. **D** PTZ + resveratrol group: Collagen accumulation was significantly reduced compared with that in the PTZ group, and the interstitial area appeared more regular. Resveratrol reduces PTZ-induced fibrosis. The collagen fibers were stained bright red/orange with Sirius Red under light microscopy. The graph presents the statistical results based on the semi-quantitative score obtained with Sirius red staining. p-values ​​represent the One-way ANOVA result, and letters above the columns indicate statistical significance according to the Tukey post-hoc test results. ^a^ vs. Sham, ^b^ vs. RSV, ^c^ vs. PTZ indicate statistical significance at the *p* < 0.05 level. Data are presented as mean ± SEM (*n* = 8)

According to the evaluations obtained with Sirius red staining, fibrosis was significantly increased in the PTZ group compared with that in all other groups. While collagen fibers were of normal morphology in the sham and RSV groups, enlarged collagen areas and separation between myocardial fibers were observed in the interstitial area in the PTZ group. The interstitial structure was more organized and collagen accumulation was reduced in the PTZ + RSV group compared with that in the PTZ group (Fig. 5). These findings support the fibrotic-reducing effect of resveratrol on PTZ-induced fibrosis. According to the Sirius Red scoring results, fibrosis was significantly increased in the PTZ group (*p* < 0.05). The fibrosis score decreased and approached the sham level in the PTZ + RSV group. Reduction of myocardial fibrosis with resveratrol was quantitatively confirmed (Fig. 5).

### Effects of resveratrol on immunofluorescent findings in rat heart tissue

**Fig. 6:**
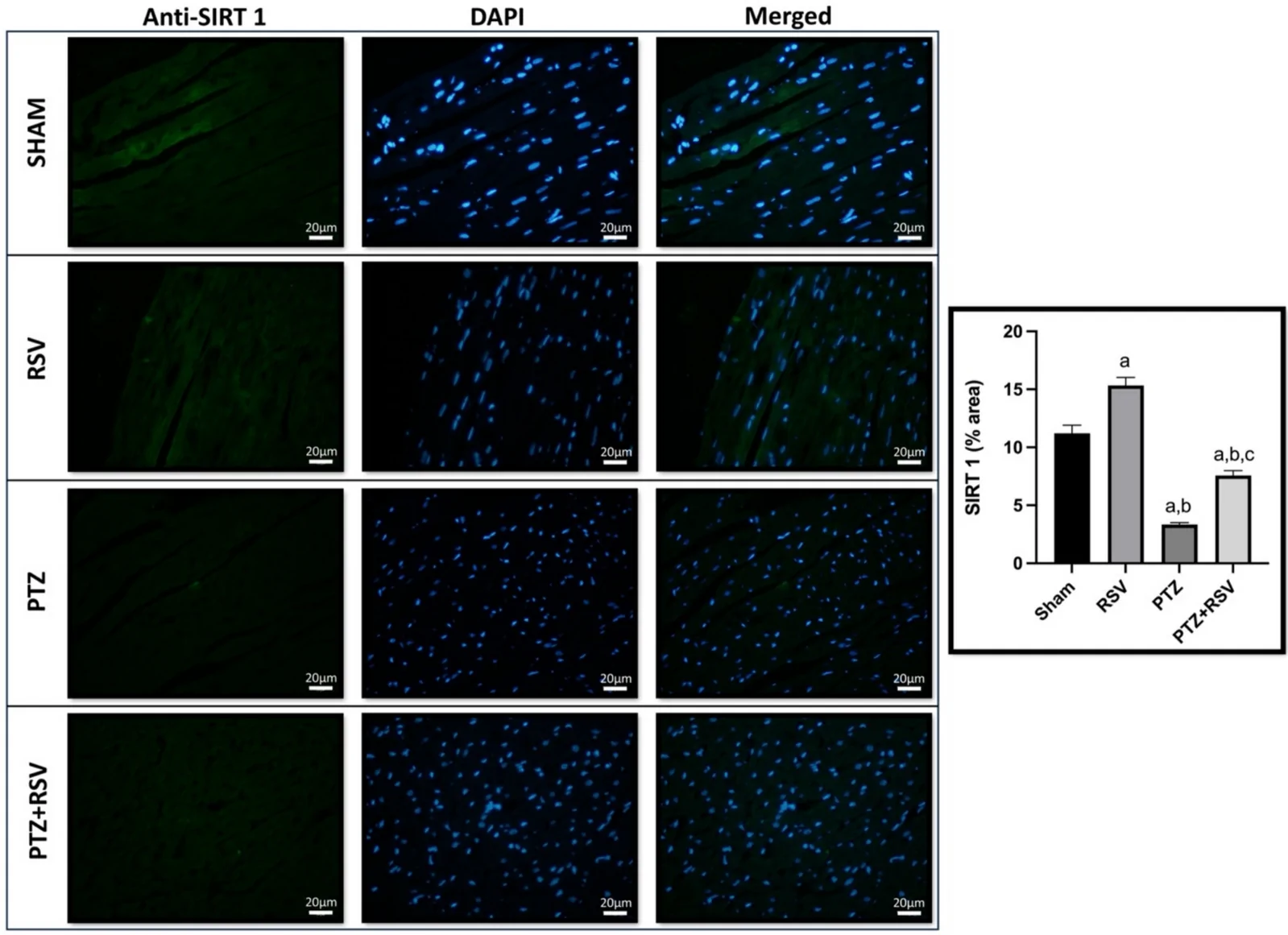
Anti-SIRT1 immunofluorescence staining images in the heart tissue (40 × magnification). FITC (green) shows anti-SIRT1-positive cells, and DAPI (blue) shows cell nuclei. The "Merge" panel represents the superimposed image of the FITC and DAPI signals. The FITC, DAPI, and Merged panels are shown for each group (Sham, RSV, PTZ, and PTZ + RSV). The graph shows the percentage area values ​​for SIRT1. ^a^ vs. Sham, ^b^ vs. RSV, ^c^ vs. PTZ indicate statistical significance at the *p* < 0.05 level

Anti-SIRT1, anti-Caspase-3, and anti-S100α levels in the heart tissue were evaluated using immunofluorescence labeling. The obtained fluorescence intensities differed between the groups. The anti-SIRT1 fluorescence signal was distinct in the sham and RSV groups and was homogeneously distributed in and around the cell nucleus. The anti-SIRT1 intensity was higher in the RSV group than in the sham group. The anti-SIRT1 signal was significantly reduced in the PTZ group, suggesting suppression of cellular stress and anti-SIRT1 activity. The anti-SIRT1 signal intensity increased again in the PTZ + RSV group, indicating that resveratrol restored anti-SIRT1 expression (Fig. 6).

**Fig. 7:**
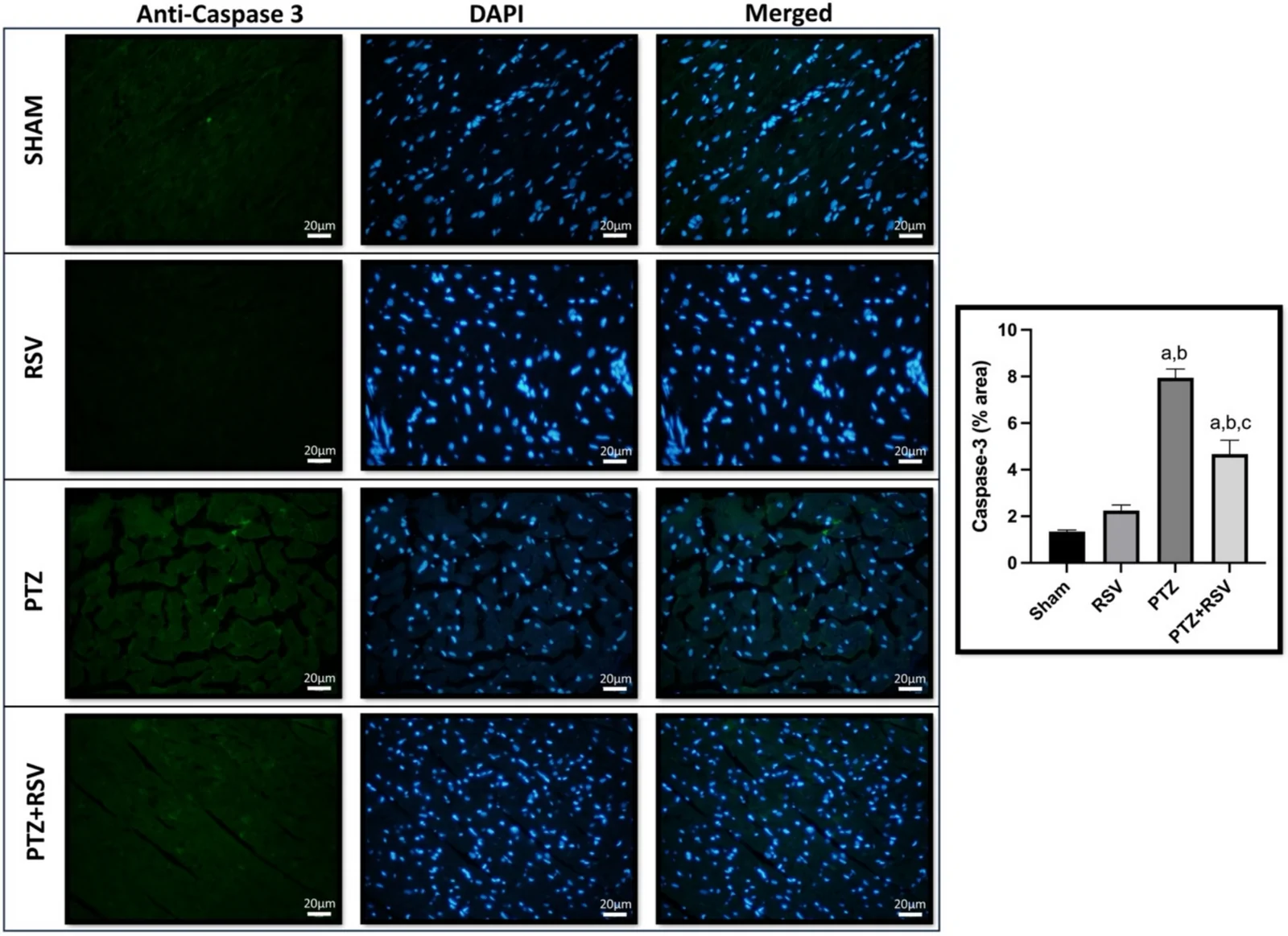
Anti-caspase-3 immunofluorescence staining images of the heart tissue (40 × magnification). Fluorescein isothiocyanate (FITC) (green) and DAPI (blue) staining indicates caspase-3-positive cells and cell nuclei, respectively. The merge panel shows the superimposed FITC and DAPI signals. For each group (sham, RSV, PTZ, and PTZ + RSV), the panel order is FITC, DAPI, and merged. The graph shows the percentage area values ​​for Caspase-3. ^a^ vs. Sham, ^b^ vs. RSV, ^c^ vs. PTZ indicate statistical significance at the *p* < 0.05 level

Low levels of anti-caspase-3-positive cells, indicative of apoptosis, were observed in the sham and resveratrol groups. The anti-Caspase-3 fluorescent signal was markedly increased in the PTZ group, with dense anti-Caspase-3-positive cells particularly in myocytes. The anti-caspase-3 signal decreased to near sham levels in the PTZ + RSV group, supporting the anti-apoptotic effect of RSV (Fig. 7).

**Fig. 8:**
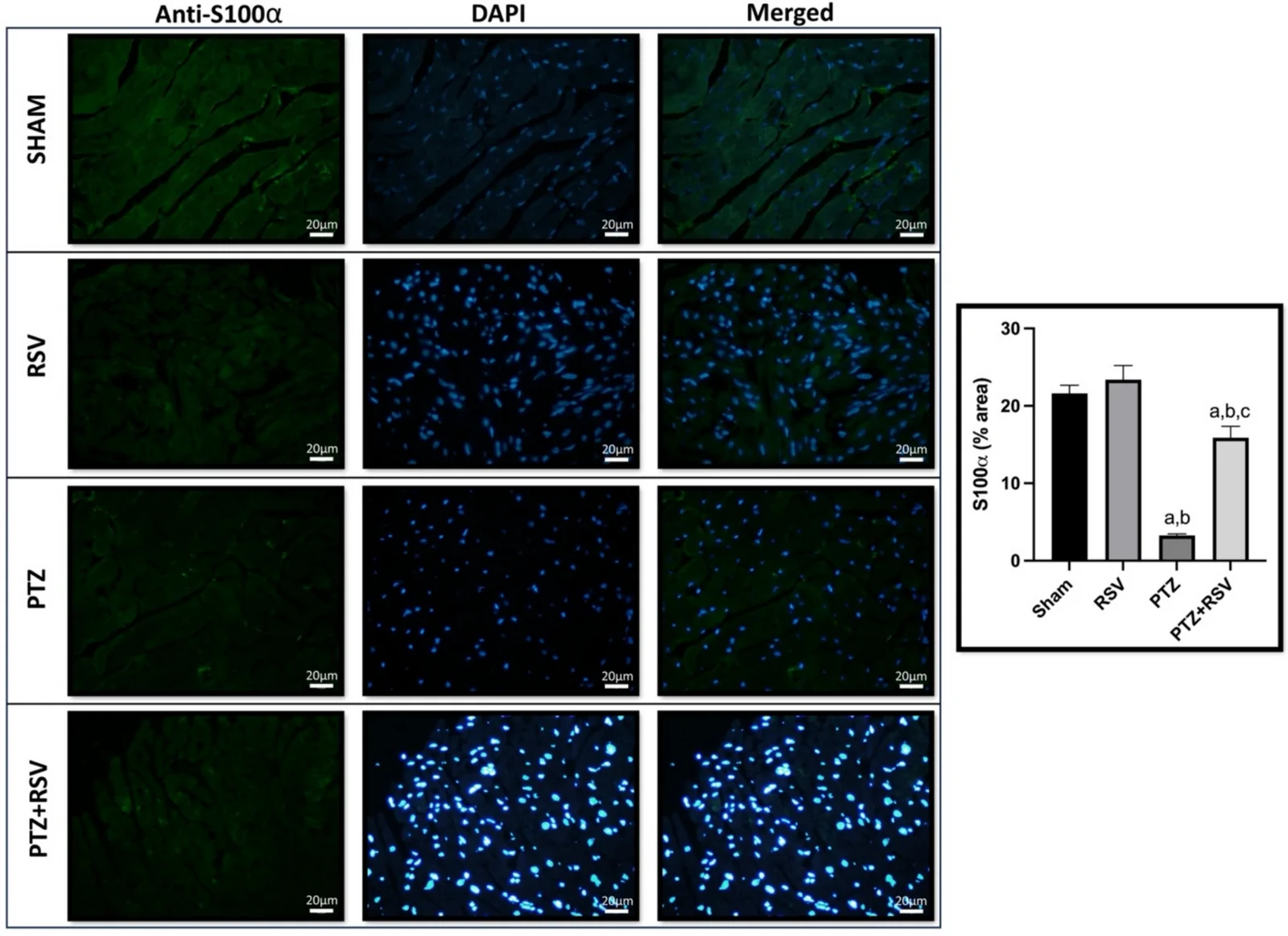
Anti-S100α immunofluorescence staining images in the heart tissue (40 × magnification). FITC (green) shows S100α-positive cells, and DAPI (blue) shows cell nuclei. The merge panel shows the FITC and DAPI signal overlays. The images correspond to the sham, RSV, PTZ, and PTZ + RSV groups. The graph shows the percentage area values ​​for S100α. ^a^ vs. Sham, ^b^ vs. RSV, ^c^ vs. PTZ indicate statistical significance at the *p* < 0.05 level

S100α immunofluorescence intensity is at physiological levels in the sham and RSV groups. S100α expression is reduced in the PTZ group, which was associated with PTZ-induced cardiac cell damage and calcium-binding protein loss. In the PTZ + RSV group, both S100α levels tended to improve and tissue architecture was restored (Fig. 8).

The SIRT1 signal was high in the sham and RSV groups, with the highest intensity observed in the RSV group. SIRT1 expression was significantly reduced in the PTZ group (*p* < 0.001). The SIRT1 level increased again in the PTZ + RSV group (*p* < 0.01 vs PTZ). This demonstrates the protective/metabolic regulatory effect of resveratrol in cardiomyocytes (Fig. 6).

The intensity of caspase-3 fluorescence increased significantly in the PTZ group (*p* < 0.001), indicating myocardial apoptosis. Resveratrol treatment significantly reduced caspase-3 signaling (*p* < 0.01 vs. PTZ) (Fig. 7).

S100α expression is decreased in the PTZ group (*p* < 0.05). This is associated with PTZ-induced cardiac cell damage and calcium-binding protein loss. In the PTZ + RSV group, S100α levels were observed to increase again, indicating the protective/stabilizing effect of RSV on cardiac cell integrity (Fig. 8).

### Effects of resveratrol on qPCR analysis and total calcium content findings in rat heart tissue

Measurements of total calcium content and qPCR revealed molecular changes in cardiac calcium homeostasis and cellular stress response in the PTZ-induced kindling model, and the impact of RSV on these changes.

### Calcium homeostasis and ion channel expressions

**Fig. 9:**
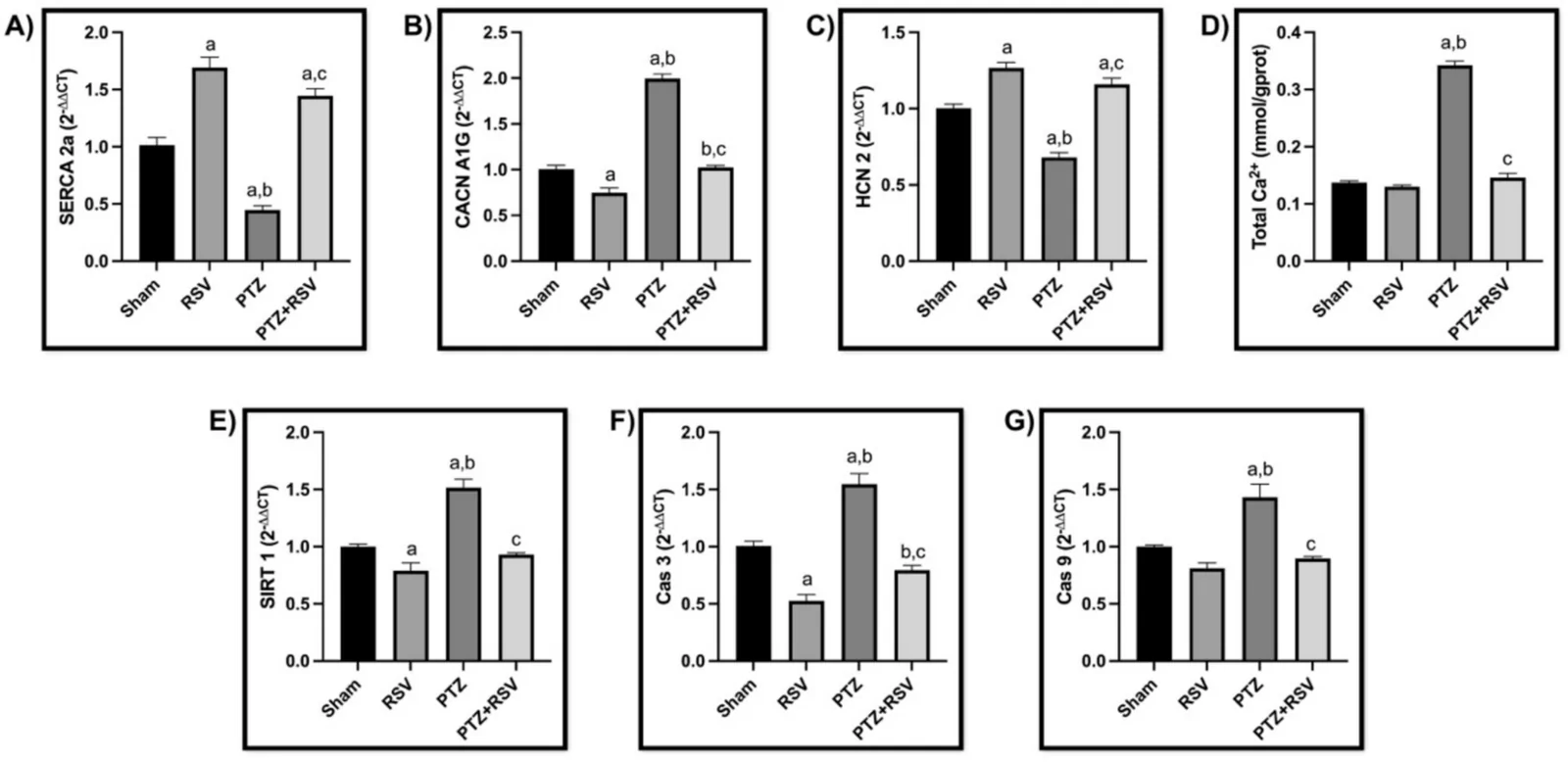
Effects of resveratrol administration on ion channels, total calcium content, apoptosis, and mRNA expression levels of genes involved in cellular stress response in heart tissue of rats kindled with pentylenetetrazol (PTZ). The figure shows, respectively, **A** SERCA 2a (ATPase Sarcoplasmic/Endoplasmic Reticulum Ca^2^⁺ Transporter 2), **B** CACN A1G (Calcium Voltage-Gated Channel Subunit Alpha1G), **C** HCN 2 (Hyperpolarization-Activated Channel 2) mRNA expression, **D** tissue Ca^2+^ levels measured by ELISA method, **E** SIRT1 (Sirtuin 1), **F** Caspase 3 (CAS3), **G** Caspase 9 (CAS9) mRNA expression. *p* values ​​represent the One-way ANOVA result, and letters above the columns indicate statistical significance according to the Tukey post-hoc test result. ^a^ vs. Sham, ^b^ vs. RES, ^c^ vs. PTZ represent statistical significance at the *p* < 0.05 level. Data are presented as mean ± SEM (*n* = 8)

PTZ Administration resulted in substantial disruptions to calcium metabolism and mRNA expression of ion channels within cardiac tissue. PTZ administration resulted in a significant decrease in SERCA 2a mRNA expression, which is essential for cardiac contraction and relaxation, compared with the sham group (*p* < 0.0001). In contrast, RSV treatment significantly elevated SERCA 2a expression in the PTZ + RSV group compared with that in the PTZ group (*p* < 0.0001) (Fig. 9A). The PTZ kindling caused a substantial increase in the mRNA expression levels of the CACNA1G subunit of the T-type calcium channel compared with the sham group (*p* < 0.0001). RSV administration in the PTZ + RSV group significantly diminished this increase compared with the PTZ group (*p* < 0.001) (Fig. 9B). The expression of HCN2 was significantly lower in the PTZ group than in the sham group, but RSV application significantly increased this reduction in the PTZ + RSV group (*p* < 0.001) (Fig. 9C). Tissue Ca + + levels, which are mediators of muscle mechanical activity, were determined using the ELISA method. The PTZ group had a significantly higher Ca^2^⁺ concentration in the heart tissue than the sham group (*p* < 0.0001). Administration of resveratrol (PTZ + RSV) resulted in a significant decrease in the high Total Ca^2^⁺ content caused by PTZ (*p* < 0.0001) (Fig. 9D).

### Apoptosis and cellular stress response gene expressions

PTZ-induced epilepsy led to considerable alterations in the mRNA expression levels of molecular markers influencing apoptosis and cellular stress response in cardiac tissue. SIRT1 mRNA expression levels were significantly higher in the PTZ group than in the sham group (*p* < 0.001). The administration of RSV (PTZ + RSV) noticeably decreased the increase caused by PTZ (*p* < 0.0001) (Fig. 9E).

The PTZ group exhibited a substantial increase in both Caspase 3 (*p* < 0.0001) and Caspase 9 (*p* < 0.001) mRNA expression levels compared with the sham group. The administration of resveratrol (PTZ + RSV) significantly decreased the expression of Caspase 3 and Caspase 9 compared with the PTZ group, with a statistical significance of *p* < 0.0001 (Fig. 9 F, G).

## Discussion

Epilepsy is a chronic neurological disorder that is strongly associated with cardiovascular comorbidities and SUDEP (Shmuely et al. 2017; Thijs et al. 2019). The pathophysiology of SUDEP is highly complex and multifactorial, involving structural cardiac damage, such as myocardial fibrosis, lethal arrhythmias and dysregulation of the autonomic nervous system (Manolis et al. 2019; Poddi et al. 2025). Using the PTZ kindling model, this study aimed to investigate the multifaceted effects of epileptogenesis on the functional, structural, and molecular properties of the heart, while evaluating the potential therapeutic efficacy of resveratrol (RSV).

Our study confirmed the effectiveness of the model by extending the seizure phase and the overall number of spikes in ECoG recordings following PTZ kindling. The treatment of RSV notably decreased the seizure phase and total spike count, in line with the anticonvulsant and neuroprotective properties of RSV observed in PTZ and kainic acid (KA) models (Gupta et al. 2002; Saha and Chakrabarti 2014; Shetty 2011; Wang et al. 2004).

Recurrent autonomic imbalances caused by chronic epileptic activity result in permanent structural damage and cellular stress in the heart (Naggar et al. 2014). Our study's results show how PTZ kindling compromises the morphological integrity and cellular survival of the heart at both the histological and immunofluorescent levels. Female rats subjected to PTZ kindling exhibited a substantial increase in heart weight and cardiomyocytes lengthening compared with the control group (Akyuz et al. 2020). This finding of hypertrophy aligns with studies indicating that autonomic changes resulting from chronic seizures impact the structural dimensions of the heart (Naggar et al. 2014). Patients with epilepsy increased sympathetic nervous activity (resulting from a sympathovagal imbalance) tend to experience cardiac remodeling (Metcalf et al. 2009a). H&E staining was used to show interstitial edema, inflammatory cell infiltration, vascular congestion, and hypereosinophilic myocytes indicative of myocardial degeneration in the PTZ group. Quantitative analysis using Sirius Red staining revealed a substantial increase in collagen accumulation, which is indicative of myocardial fibrosis. Increased fibrosis stiffens the myocardium and interferes with electrical conduction, making it a conducive environment for arrhythmias (Poddi et al. 2025; Weber and Brilla 1991). The structural damage is consistent with the structural pathologies found in patients with SUDEP (Jansen and Lagae 2010; Mir et al. 2025). Treatment for RSV helped preserve the structure's integrity by substantially lowering the fibrosis score and reducing histological degeneration. The immunofluorescence results of our research demonstrated that epileptic damage impacted cell survival in the heart through the SIRT1 and Caspase-3 pathways. Cells positive for Caspase-3 (FITC signal) that are involved in the final stage of apoptosis showed a significant increase in the PTZ group, with a strong anti-Caspase-3 signal observed, especially in cardiac muscle cells. PTZ kindling-induced cardiac cell death was demonstrated by this finding. The observed outcome aligns with existing research findings (Bengzon et al. 1997; Rzayev et al. 2022; Zhang et al. 1998) which indicate that triggering epilepsy (either with PTZ or Status Epilepticus) leads to apoptosis in the brain's hippocampus region, characterized by Caspase-3 activation and DNA fragmentation, and also demonstrates that this apoptotic process can extend to the heart muscle. The administration of RSV (a combination of PTZ and RSV) resulted in a significant decrease in Caspase-3 fluorescence compared with the PTZ group, thus validating the potent anti-apoptotic properties of RSV. Significant reductions in fluorescent signal intensity of the anti-SIRT1 protein, a key metabolic regulator and protective protein, were observed in the PTZ group relative to the sham group, indicating that PTZ kindling inhibited cellular stress and SIRT1 function. The administration of RSV (combined with PTZ) restored the intensity of the anti-SIRT1 signal and resulted in a significant recovery compared with the PTZ group alone. The findings from existing research are in line with the notion that RSV activates SIRT1, offering protection against neurodegenerative diseases by regulating metabolic function (Baur and Sinclair 2006; Pallàs et al. 2009; Shetty 2011). A reduction in S100α immunofluorescence intensity was observed in the PTZ group. S100α is related to calcium-binding proteins, and its reduced expression has been associated with PTZ-induced cardiac cell damage and protein loss. In the PTZ + RSV group, S100α levels were observed to increase again, indicating the protective/stabilizing effect of RSV on cell integrity. Our study has shown that epileptic damage significantly impairs heart function by closely investigating how PTZ kindling affects the natural contractions of the heart, as measured through a series of frequency-dependent isometric contraction tests (ranging from 1 to 5 Hz) in a controlled, isolated environment. In the PTZ group, CF and AUC values exhibited notable declines relative to the sham group at every stimulation frequency from 3 to 5 Hz. Epileptic damage typically diminishes the myocardium's ability to contract and reduces its systolic capacity, which agrees with studies showing that epileptic activity directly harms cardiac cells. Metcalf et al. (2009) observed cardiac myofilament damage and heightened risk of arrhythmia in rats subjected to Status Epilepticus (SE) (Metcalfet al. 2009b). Direct damage to myofilaments directly causes a loss of CF/AUC by reducing the effectiveness of actin-myosin cross bridges (Metcalf et al. 2009b). Our study's findings of a substantial increase in myocardial fibrosis detected through histological examination lead to a decrease in the number of myocytes contributing to contraction and a weakening of contraction force due to the introduction of stiff connective tissue between myocardial fibers (Weber and Brilla 1991). Consequently, the results of mechanical loss in our study are in line with both degeneration at the cellular level and fibrosis at the macro level, as well as reports on cardiac damage associated with epilepsy in general. PTZ kindling-induced kinetic abnormalities showed that diastolic function was severely impaired. In our study, contraction and relaxation velocity parameters decreased in the PTZ group compared with the Sham group at all frequencies except 1 Hz. This finding strongly suggests that cytosolic calcium clearance is slowed, which may primarily be driven by impaired calcium reuptake into the Sarcoplasmic Reticulum (SR) due to downregulated SERCA 2a. However, it is important to acknowledge that a deficiency or functional alteration in the Na +/Ca^2^⁺ exchanger (NCX) could also contribute to these delayed relaxation kinetics. Notable prolongation of CT scans was observed in the PTZ group, especially at frequencies of 4 and 5 Hz. This prolongation indicates that myocardial relaxation is delayed and cytosolic calcium clearance is impaired. The molecular findings of our study align with these diastolic dysfunction results. Furthermore, while the isolated papillary muscle model provides highly valuable insights into the intrinsic mechanical contractility of the myocardium, it has inherent limitations when extrapolating these specific ex-vivo mechanical findings to overall global ventricular and in-vivo cardiac function.

Our study discovered a considerable drop in SERCA 2a mRNA expression; this latter enzyme is the key pump governing the relaxation process. This reduction in expression directly accounts for the decrease in -dF/dt and CT prolongation at the molecular level. Studies of the hippocampus also indicate that epilepsy causes cytosolic calcium build-up and triggers endoplasmic reticulum (ER) stress (Hernández-Fonseca and Massieu 2005). Increased fibrosis in the heart muscle impairs diastolic filling by lowering the elasticity of the heart during relaxation and thereby indirectly contributing to prolonged CT times (Weber and Brilla 1991). Reports have been made of increased left ventricular stiffness and diastolic dysfunction in individuals with epilepsy, even when there is no obvious cardiovascular disease (Naggar et al. 2014; Shmuely et al. 2017). The isolated organ recordings in our study provide strong evidence that this diastolic defect is physiologically present in the PTZ kindling model. The prevailing view in current literature and sources suggest that epilepsy has a negative impact on cardiac contraction and relaxation rates. Echocardiographic measurements were conducted by Naggar et al. (2014) in the KA-SE model (Naggar et al. 2014) and revealed structural alterations. However, the comprehensive mechanical kinetic analysis that isolated organ recordings provide in this study; nonetheless, the findings confirm that long-term epileptic status has a detrimental impact on the heart. Compared with the sham group, PTZ kindling significantly decreased the expression of SERCA 2a mRNA, the primary protein regulating relaxation quality. SERCA 2a deficiency reduces SR-induced Ca^2+^ reuptake. Prolonged slowing of the process leads to an increased concentration of Ca^2+^ ions in the cytosol, which accounts for the prolongation of CT and the reduction in -dF/dt (relaxation defect) at the molecular level. The substantial increase in total Ca^2^⁺ content noted in the PTZ group stems from cellular imbalance caused by decreased SERCA and heightened Ca^2^⁺ influx (augmented CACN A1G). Calcium overload is toxic to heart muscle cells and results in permanent cellular damage (Hernández-Fonseca and Massieu 2005). In our study, the observed decrease in CF and AUC parameters is directly linked to this overload compromising the structural integrity of actin-myosin myofilaments (Metcalf et al. 2009b). Increasing fibrosis decreases the number of contraction units, reducing the mechanical force.

A significant reduction in HCN2 mRNA expression was observed in the PTZ group. This result is in line with that of Powell et al. (2014), who noted a decrease in HCN2 expression in both acquired and genetic epilepsy models. While HCN4 is the predominant isoform responsible for pacemaker activity in the sinoatrial node, HCN2 is the major isoform expressed in ventricular cardiomyocytes. Therefore, the decrease in HCN2 levels observed in our ventricular samples likely causes a defect in the local pacemaker current (If). Although a more robust assessment of central pacemaker dysfunction would require analysis of sinoatrial node tissue, this ventricular HCN2 deficiency increases the intrinsic risk of abnormal heart rhythms and ventricular arrhythmogenesis. The decrease in HCN2 expression is consistent with a 22% reduction in HCN2 protein expression in rat hearts following SE induced by KA. HCN channels have a significant impact on QTc interval modulating (Powell et al. 2014). Decreases in mRNA and protein expression of inwardly rectifying potassium (Kir) channels, like Kir2.x and Kir3.x, have been observed in the PTZ kindling model (Akyüz et al. 2018). The suppression of Kir2.x contributes to the repolarization imbalance by prolonging the potent action (Surges et al. 2010). These findings indicate the presence of repolarization abnormalities in the PTZ model, in line with clinical findings such as prolonged QTc interval. Reports of decreased vulnerability to ventricular fibrillation in certain models of epilepsy in rats suggest that systemic factors, including respiratory and autonomic imbalance, are more closely linked to the cause of SUDEP than intrinsic arrhythmic susceptibility alone (Bateman et al. 2008). Our research showed that PTZ-induced kindling led to a substantial increase in the mRNA expression of the CACN A1G gene (T-type Ca^2+^ channel). An increase in T-type Ca^2+^ channels may contribute to a calcium overload (Demirtaş Şahin et al. 2020).

Treatment of RSV was found to offer multi-faceted protection against cardiac dysfunction in epilepsy patients. RSV application restored SERCA 2a and reduced cellular stress, improving isometric contraction parameters (CF/AUC and ± dF/dt), thereby restoring myocardial function and preserving structural integrity by lowering myocardial fibrosis scores. RSV has beneficial effects on intracellular calcium dynamics and suggests a protective pathway consistent with the neuroprotective effects of agents that reduce PTZ-induced Ca^2^⁺ overload and associated ER stress, such as agents like 4-PBA (Rzayev et al. 2022). Dysregulation of HCN and T-type Ca^2^⁺ channels has been linked to SUDEP (Powell et al. 2014), and the molecular level regulation by RSV is thought to lower the risk of arrhythmias. In line with its role in metabolic regulation, RSV reversed the suppression of anti-SIRT1 protein signaling caused by PTZ (Shetty 2011). The activation of SIRT1 controls inflammation, cell death, and metabolic processes (Chung et al. 2010). RSV also significantly decreased Caspase 3/9 mRNA expression and Caspase-3 protein activity, thus inhibiting apoptosis caused by calcium overload RSV's anti-apoptotic effects have been extensively shown in the literature; for instance, RSV decreases neuronal damage and apoptosis in models of traumatic brain injury (Liu et al. 2011; Singleton et al. 2010) and safeguards against cell death caused by oxidative stress (Akkoca et al. 2025; Jang and Surh 2003; Nicolini et al. 2001; Shetty 2011). The neuroprotective effect of RSV is also related to its potent antioxidant characteristics (Baur and Sinclair 2006; Shetty 2011). RSV decreases the incidence of seizures and oxidative stress caused by KA, indicating that RSV indirectly prevents caspase activation by neutralizing the oxidative damage triggered by PTZ kindling (Saha and Chakrabarti 2014).

Our study has certain limitations. To evaluate the direct intrinsic mechanical contractility of the myocardium independent of autonomic nervous system fluctuations, we utilized isolated papillary muscle recordings in an organ bath. While this approach clearly demonstrates cellular and mechanical damage at the organ level, the lack of in-vivo electrocardiography (ECG) measurements limits our ability to observe in-vivo arrhythmogenesis (e.g., QT dispersion or Heart Rate Variability), which is directly associated with SUDEP. Future studies are planned to utilize in-vivo telemetric ECG to correlate the intrinsic mechanical and molecular defects identified here with in-vivo arrhythmias.

## Conclusion

Our research ultimately uncovers the underlying mechanism of heart failure linked to the risk of SUDEP triggered by PTZ kindling. Recordings of isolated papillary muscle contraction revealed significant mechanistic dysfunction in the PTZ group; this was characterized by significant decreases in CF, AUC, and ± dF/dtmax parameters at 2, 3, 4, and 5 Hz frequencies, and an increase in CT duration under high-frequency stimulation. The molecular basis for these functional losses is a substantial drop in the expression of SERCA 2a mRNA, the main pump responsible for relaxation, and the resulting excessive burden of total Ca^2+^ content. The sharp decrease in frequency responses observed in the PTZ group in our study indicates that the heart is insufficient in situations requiring high metabolic demands after a seizure. ATP consumption accelerates during high-performance work, and ATP reduction slows down sarcoplasmic/endoplasmic reticulum Ca2 + -ATPase (SERCA) pumps and the cross-bridge loop, increasing the muscle's susceptibility to fatigue (Cadile et al. 2023). Impairments in Ca2 + release and reuptake in the sarcoplasmic reticulum also lead to impairments in muscle mechanical activity (Smith-Blair 2002). Treatment of RSV significantly lowered the severity of seizures and the overall number of spikes, restored suppressed SERCA 2a expression, and reduced Ca^2^⁺ overload, thereby ameliorating myocardial dysfunction by correcting abnormalities in isometric contraction parameters. RSV protected both structural and cellular integrity by significantly attenuating PTZ-induced myocardial fibrosis and apoptosis, which was indicated by Caspase-3/9 activation, stabilizing ion channel abnormalities through improved CACNA1G and HCN2 expression and increasing SIRT1 protein levels. The findings suggest that RSV has the potential to serve as a supportive or adjuvant therapeutic agent against cardiac injury associated with SUDEP risk through various pathways.

## Supplementary Information

Below is the link to the electronic supplementary material.

ESM 1 (XLSX 13.6 KB)

ESM 2 (DOCX 194 KB)

## Data Availability

All source data for this work (or generated in this study) are available upon reasonable request.
