# Supplementary material for: Epilepsy-induced heart damage and sudep risk: ion channelopathies, calcium homeostasis, and the therapeutic potential of resveratrol
Source: Naunyn Schmiedebergs Arch Pharmacol. 2026 Jun 10;399(11):18365–82. doi: 10.1007/s00210-026-05557-6 (PMC13503426; doi:10.1007/s00210-026-05557-6)
Supplement: Supplementary file 1 [file 210_2026_5557_MOESM2_ESM.docx]

**Supplementary Figure 1.** Intergroup comparison of parameters obtained from isometric contraction recordings of rat papillary muscles. Isometric contractility data were analyzed using the Kruskal-Wallis test followed by Dunn's multiple comparison test. ^a^ vs. Sham, ^b^ vs. RSV, ^c^ vs. PTZ indicate statistical significance at the p<0.05 level.
